# From detection to decision: a conceptual framework of uncertainty migration in disorders of consciousness

**DOI:** 10.3389/fneur.2026.1940448

**Published:** 2026-09-09

**Authors:** Fangting Wang, Yufei Xue, Haibo Di

**Affiliations:** 1 International Institute for Vegetative State and Consciousness Science, Hangzhou Normal University, Hangzhou, China; 2 Zhejiang-Belgium Joint Laboratory for Disorders of Consciousness, Hangzhou Normal University, Hangzhou, China; 3 School of Public Health and Nursing, Hangzhou Normal University, Hangzhou, China; 4 School of Basic Medical Sciences, Hangzhou Normal University, Hangzhou, China

**Keywords:** clinical decision-making, consciousness assessment, disorders of consciousness, multimodal neurotechnology, uncertainty

## Abstract

Accurate assessment of consciousness in patients with disorders of consciousness (DoC) remains a longstanding challenge in neurocritical care and neurorehabilitation. Although standardized behavioral assessments continue to serve as the clinical foundation for diagnosis, their reliance on overt behavioral responses may underestimate residual consciousness in a subset of patients. Recent advances in multimodal neuroimaging and neurophysiological techniques have substantially expanded the capacity to detect covert consciousness, reducing some of the inherent limitations of behavioral assessment and facilitating the identification of patients with preserved cognitive processing despite an absence of observable responses. However, these technological advances have not eliminated uncertainty in DoC assessment. Instead, uncertainty may be reconfigured as assessment progresses from behavioral observation to neural measurement, the inference of conscious awareness, prognostic evaluation, and clinical decision-making. Whether neural activity can reliably reflect subjective conscious experience, how neurophysiological evidence should inform prognostic evaluation and therapeutic decisions, and the emerging risks introduced by artificial intelligence-assisted assessment all indicate that uncertainty in DoC has evolved beyond a purely technical challenge into a broader problem of interpretation and clinical decision-making. Artificial intelligence (AI) further intersects with these uncertainties across multiple stages by contributing to signal processing, multimodal integration, consciousness inference, prognostic prediction, and clinical decision support. In this review, we propose the conceptual framework of “uncertainty migration” to characterize how uncertainty may change in form, persist across epistemic transitions, be redistributed between stages or actors, and accumulate through interactions between residual and newly introduced uncertainties across five interconnected domains of DoC assessment: behavioral assessment, neural measurement, consciousness inference, prognostic evaluation and clinical decision-making. These processes of transformation, propagation, redistribution, and accumulation are neither mutually exclusive nor necessarily linear, as discordant multimodal findings and longitudinal changes may prompt the reassessment of earlier evidence. We argue that multimodal approaches fundamentally change how consciousness-related evidence is acquired, and may reduce particular sources of uncertainty without eliminating the inherently inferential nature of consciousness assessment. Consequently, future advances in the DoC field should extend beyond improving diagnostic accuracy toward developing comprehensive strategies for uncertainty management that emphasize rigorous interpretation of evidence, longitudinal reassessment, multimodal evidence integration, transparent AI governance,and shared decision-making. Such an approach may better support cautious, transparent, and patient-centered clinical decisions in the face of persistent uncertainty.

## 1 Introduction

Disorders of consciousness (DoC) represent one of the most challenging clinical conditions in neurocritical care and neurorehabilitation (1). Encompassing coma, unresponsive wakefulness syndrome (UWS), and minimally conscious state (MCS), DoC present substantial challenges for the accurate assessment of consciousness, which is fundamental to establishing diagnosis, informing prognostic evaluation, and guiding therapeutic decision-making. Despite decades of research, determining whether patients retain residual consciousness in the absence of reliable behavioral responses remains one of the central unresolved questions in the field. The uncertainty arising from this problem is not confined to diagnosis but may also shape prognostic evaluation and clinical decision-making throughout the continuum of care.

At present, standardized behavioral assessment remains the cornerstone of DoC diagnosis, with the Coma Recovery Scale-Revised (CRS-R) being widely used as the clinical reference standard (2). Nevertheless, behavioral assessments rely heavily on the patient’s ability to produce observable behavioral responses and are therefore susceptible to confounding factors such as motor impairment, fluctuations in arousal, and the effects of sedative medications, potentially leading to an underestimation of residual consciousness (3). The recognition of cognitive motor dissociation (CMD) has further challenged the assumption that the absence of behavioral responsiveness necessarily indicates the absence of consciousness. A subset of behaviorally unresponsive patients has been shown to exhibit command-following neural activity despite lacking observable motor output, indicating that behavioral unresponsiveness cannot necessarily be equated with unconsciousness. These findings underscore the limitations of behavioral assessment alone and highlight the need for complementary approaches capable of detecting covert consciousness (4).

To address these limitations, multimodal neurotechnologies have increasingly been incorporated into the assessment of DoC. Functional magnetic resonance imaging (fMRI), electroencephalography (EEG), positron emission tomography (PET), and brain-computer interface (BCI)-based approaches enable the detection of neural signatures potentially associated with residual consciousness that may not be captured by behavioral assessment alone. By expanding access to consciousness-related evidence beyond observable behavior, these techniques have broadened the possibilities for identifying covert consciousness and may contribute to improved diagnostic accuracy.

Despite the substantial advances achieved by multimodal neurotechnologies, existing research has primarily focused on improving diagnostic sensitivity and identifying covert consciousness, while paying comparatively little attention to whether these technological advances fundamentally reduce uncertainty in DoC assessment (5). In practice, as the focus of assessment has progressively expanded from behavioral observation to include the detection and interpretation of neural activity, the central challenge has likewise extended beyond determining whether consciousness can be detected to understanding how neural evidence should be interpreted and translated into prognostic evaluation and clinical decision-making. Consequently, improved detection capabilities do not necessarily eliminate uncertainty; they may instead transform or redistribute it across the assessment process.

To conceptualize this evolving pattern, we introduce the framework of “uncertainty migration.” We define uncertainty migration as the dynamic process through which uncertainty may change in form, persist across epistemic transitions, shift between components or actors, and interact with newly introduced uncertainties across an assessment pathway encompassing behavioral assessment, neural measurement, consciousness inference, prognostic evaluation, and clinical decision-making. Within this framework, transformation, propagation, redistribution, and accumulation constitute related but analytically distinct mechanisms. Transformation occurs when uncertainty changes form as evidence is translated from one epistemic level to another—for example, when uncertainty about the meaning of an absent behavioral response becomes uncertainty about the significance of a detected neural signal. Propagation refers to the persistence of unresolved uncertainty from an earlier stage and its continued influence on subsequent inferences or decisions. Limitations in signal acquisition, for instance, may constrain the confidence that can later be placed in consciousness attribution and prognosis. Redistribution occurs when an assessment method reduces uncertainty in one component of the pathway while shifting the remaining uncertainty toward another component, stage, or actor. Multimodal neurotechnologies may reduce dependence on motor responsiveness while increasing the interpretive burden placed on clinicians and researchers. Accumulation occurs when residual uncertainty inherited from earlier stages interacts with uncertainty newly introduced at later stages, potentially broadening the range of plausible interpretations or clinical decisions rather than progressively narrowing it.

These mechanisms are not mutually exclusive, and their operation is not necessarily linear. A single transition may simultaneously transform one form of uncertainty, propagate unresolved limitations, redistribute interpretive responsibility, and contribute to the accumulation of uncertainty. Moreover, discordant multimodal findings and longitudinal changes may prompt reassessment of earlier evidence, producing feedback loops through which uncertainty is repeatedly recalibrated across the assessment pathway. Thus, uncertainty migration does not imply that uncertainty simply moves intact from one stage to the next, nor that earlier uncertainty is eliminated and replaced. Rather, it describes how uncertainty is dynamically reconfigured as different forms of evidence are generated, interpreted, and translated into clinical judgment.

Building on previous reviews of the limitations of behavioral assessment and the diagnostic performance of multimodal neurotechnologies (4, 6), this review develops uncertainty migration as a conceptual-analytic framework for examining how uncertainty is reconfigured across the DoC assessment pathway. The contribution of this review is threefold. First, we synthesize the major forms of uncertainty identified across the existing literature and organize them into an analytical taxonomy encompassing observational, measurement, inferential, prognostic, normative, and algorithmic uncertainty. Second, we analytically distinguish four interrelated processes: transformation, propagation, redistribution, and accumulation, to clarify how these forms of uncertainty may interact across behavioral assessment, neural measurement, consciousness inference, prognostic evaluation, and clinical decision-making. We also incorporate feedback processes through which discordant or longitudinal findings may prompt the reassessment and recalibration of earlier evidence. Third, we draw out the implications of this framework for uncertainty management in clinical practice, emphasizing rigorous evidence interpretation, repeated and longitudinal reassessment, multimodal evidence integration, explicit recognition of discordance, and shared decision-making under conditions of persistent uncertainty.

**Figure 1:**
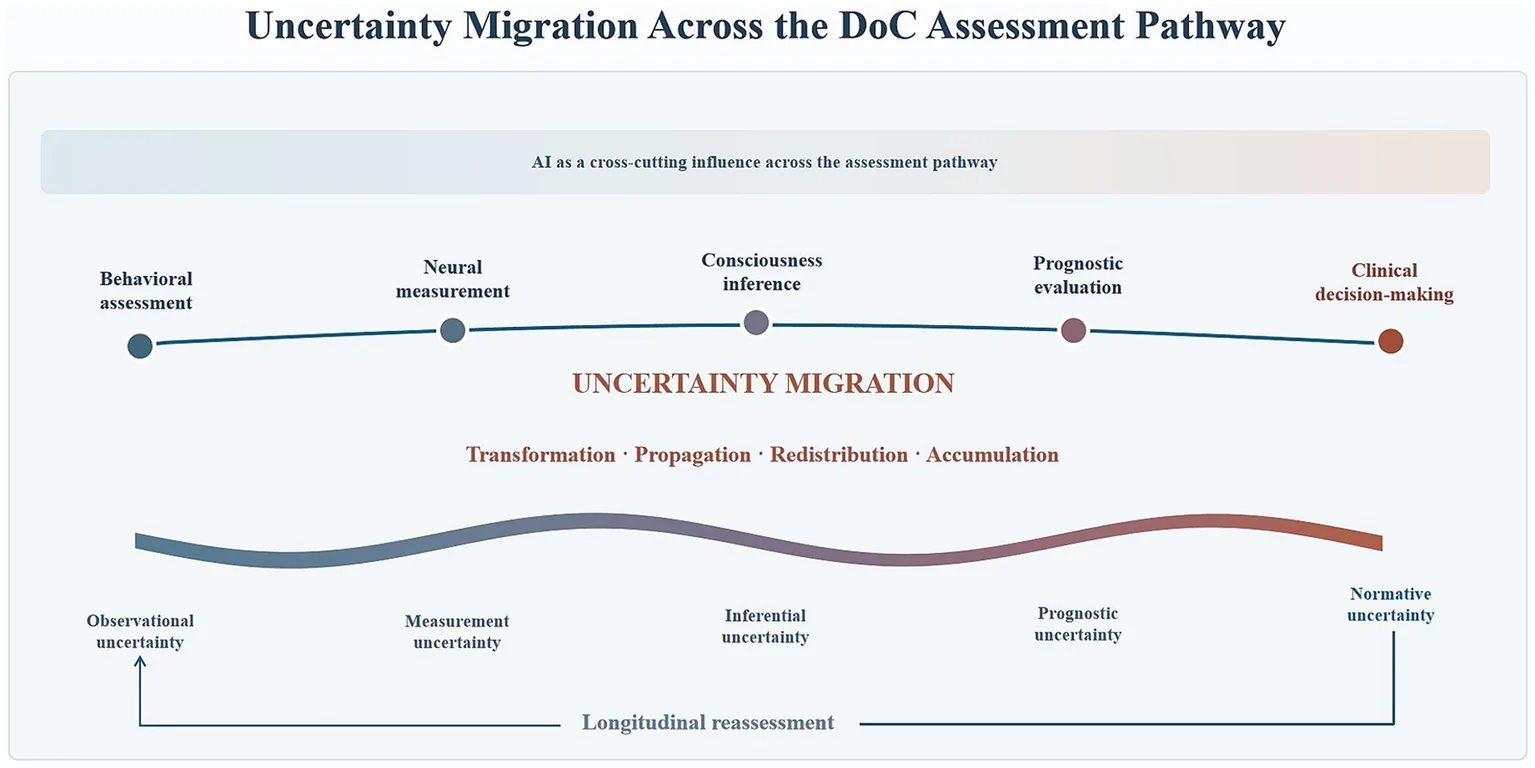
Uncertainty migration across the disorders of consciousness assessment pathway. Uncertainty may be transformed, propagated, redistributed, or accumulated as evidence progresses across five interconnected domains: behavioral assessment, neural measurement, consciousness inference, prognostic evaluation, and clinical decision-making. Artificial intelligence operates as a cross-cutting layer across the assessment pathway, while longitudinal reassessment provides feedback to earlier stages in response to discordant findings or changes over time.

Based on this perspective, Figure 1 presents uncertainty migration as a dynamic and potentially iterative process across five interconnected domains of DoC assessment: behavioral assessment, neural measurement, consciousness inference, prognostic evaluation, and clinical decision-making. The framework illustrates how uncertainty may persist and propagate across stages, change in form through transformation, be redistributed across different components or stages of the pathway, and accumulate as residual uncertainty interacts with uncertainty introduced at later stages. The feedback pathway represents the longitudinal reassessment of earlier behavioral and neural evidence in response to discordant multimodal findings or changes over time. AI is represented as a cross-cutting layer because algorithmic methods can influence signal processing, multimodal integration, consciousness-state inference, prognostic prediction, and decision support across multiple domains rather than operating exclusively at the final decision stage. The following sections examine the principal forms of uncertainty within each domain and the mechanisms through which they migrate across the assessment pathway.

## 2 Observational uncertainty in behavioral assessment

### 2.1 Clinical value of behavioral assessment

Consciousness is generally considered to comprise two fundamental dimensions: arousal and awareness. In clinical practice, however, these dimensions do not always coexist. Some patients may exhibit preserved arousal in the absence of overt awareness, as exemplified by UWS, whereas others retain limited but discernible awareness, with conscious behaviors that are often subtle, intermittent, or inconsistent, as observed in MCS (6). Because subjective conscious experience cannot be measured directly in clinical settings, clinicians must infer consciousness from observable behavioral responses. Consequently, behavioral assessment has long remained the cornerstone of diagnosis in patients with disorders of consciousness.

At present, standardized behavioral assessment remains the foundation of clinical diagnosis in patients with DoC, with the CRS-R widely regarded as the clinical reference standard (7). Compared with neuroimaging and neurophysiological techniques, behavioral assessment offers several practical advantages, including ease of administration, low cost, and the feasibility of repeated bedside evaluation, making it applicable across diverse healthcare settings. Furthermore, structured serial assessments can substantially improve diagnostic consistency and reliability by capturing fluctuations in behavioral responsiveness over time (8).

Nevertheless, behavioral assessment does not directly measure consciousness itself but rather evaluates whether consciousness can be expressed through observable behavior. Consequently, its clinical value has always been accompanied by inherent methodological limitations.

### 2.2 Limitations of behavior-dependent assessment

Despite its substantial clinical value, the principal limitation of behavioral assessment lies in its reliance on observable behavioral responses. Previous studies have reported misdiagnosis rates of approaching 40% among patients with DoC, suggesting that behavioral responsiveness alone may not reliably reflect the patient’s true level of consciousness (3). Importantly, such misdiagnosis cannot be attributed solely to inadequate assessment procedures or limited clinical experience; rather, it reflects a fundamental methodological constraint of behavioral assessment, namely the assumption that preserved consciousness can be reliably inferred from observable behavior. In reality, patients with DoC frequently present with severe motor impairment, sensory pathway dysfunction, language deficits, and fluctuations in arousal. Consequently, even when some degree of consciousness is preserved, they may be unable to generate consistent and detectable behavioral response (9). Furthermore, because current neurobehavioral diagnostic criteria require observable evidence of conscious behavior, covert consciousness may remain undetected when motor, language, or motivational impairments prevent its behavioral expression, leading to an underestimation of the patient’s actual level of consciousness.

A major turning point in recognizing the limitations of behavioral assessment was the emergence of the concept of CMD. Neuroimaging studies demonstrated that some behaviorally unresponsive patients were nevertheless able to generate command-following neural activity despite the absence of observable motor responses, a phenomenon subsequently conceptualized as CMD (10, 11). These findings stimulated growing interest in covert consciousness and fundamentally challenged the long-standing assumption underlying conventional behavioral assessment-that the absence of behavioral responsiveness necessarily indicates the absence of consciousness.

Importantly, CMD does not constitute direct evidence of subjective conscious experience; rather, it demonstrates that the absence of behavioral evidence cannot be simply equated with the absence of consciousness. In other words, the key implication of CMD is not that it proves the presence of consciousness, but that it reveals the limitations of behavioral assessment in detecting residual consciousness. This finding has prompted a reconsideration of the assumption that behavioral responsiveness and consciousness are always tightly coupled and has served as an important impetus for the development of multimodal neurotechnologies.

### 2.3 Challenges in assessing the dynamic nature of consciousness

Beyond the diagnostic uncertainty arising from reliance on behavioral responses, the intrinsically dynamic nature of consciousness further complicates the assessment of DoC. Evidence indicates that some patients exhibit substantial fluctuations in behavioral responsiveness even within a single day, displaying signs of conscious behavior at certain time points while showing no detectable responses at others (12, 13). Consequently, the outcome of a single behavioral assessment may be strongly influenced by transient changes in arousal, increasing the risk of diagnostic inaccuracy. These findings underscore the importance of repeated and longitudinal behavioral assessments rather than relying on isolated examinations.

The dynamic nature of consciousness is also reflected in the prolonged trajectory of recovery following severe brain injury. Accumulating evidence suggests that recovery of consciousness is not confined to the acute or subacute stages but may continue over several years. Traditional clinical perspectives have generally assumed that the likelihood of recovery declines substantially beyond 1 year after injury, particularly in patients with UWS (14). However, a mega-analysis including 3,290 patients demonstrated that the plateau of the Kaplan–Meier recovery curve occurred considerably later than previously anticipated, with annual rates of recovery of consciousness remaining between 34 and 39% regardless of disease duration (15). These findings suggest that the temporal dynamics of recovery are substantially more complex than traditionally assumed, highlighting the need for continued reassessment throughout the course of DoC.

Overall, although behavioral assessment remains the cornerstone of clinical diagnosis in patients with DoC, its reliance on observable behavioral responses inherently limits its ability to accurately reflect the patient’s true state of consciousness. Restricted behavioral expression, cognitive motor dissociation, temporal fluctuations in consciousness, and the potential for delayed recovery collectively constitute major sources of observational uncertainty in DoC assessment. Against this background, neuroimaging and neurophysiological techniques have increasingly been adopted to overcome the limitations of behavioral observation by providing complementary evidence of consciousness, thereby driving the development of multimodal assessment approaches.

The transition from behavioral observation to neural measurement illustrates an initial transformation and redistribution of uncertainty. Uncertainty about whether an absent behavioral response reflects unconsciousness or an impaired capacity for behavioral expression is not fully resolved by introducing neuroimaging or neurophysiological assessment; instead, it is partly transformed into uncertainty about how neural evidence should be acquired and interpreted. At the same time, reducing reliance on observable motor responses redistributes the evidential burden from the detection of behavioral signs toward the evaluation of neural evidence. Observational uncertainty may also propagate into subsequent assessment because multimodal neurotechnologies provide complementary rather than substitutive evidence, while fluctuations in arousal and limitations in behavioral expression continue to require repeated behavioral assessment. Thus, the introduction of multimodal neurotechnologies does not eliminate uncertainty but reconfigures its form and distribution as the assessment pathway moves from behavioral observation to neural measurement.

## 3 Measurement uncertainty in multimodal neurotechnologies

### 3.1 Clinical value of multimodal assessment

As the limitations of behavioral assessment have become increasingly apparent, growing efforts have been directed toward identifying residual consciousness through neural activity rather than relying exclusively on observable behavioral responses. Over the past two decades, multimodal neurotechnologies-including fMRI, EEG, PET, and transcranial magnetic stimulation combined with electroencephalography (TMS-EEG)—have been increasingly incorporated into the assessment of patients with DoC and have emerged as a major focus of contemporary research. Consistent with this trend, a survey of healthcare professionals found that more than 70% of respondents considered bedside behavioral assessment alone insufficient for accurately evaluating patients with DoC, while nearly 60% regarded advanced neurotechnologies as an essential component of optimal consciousness assessment (16).

The clinical value of multimodal assessment lies not merely in expanding the range of diagnostic techniques but in providing complementary evidence beyond behavioral observation. Active paradigms assess a patient’s ability to intentionally modulate neural activity in response to commands, whereas passive paradigms evaluate preserved neural processing of linguistic, auditory, or emotionally salient stimuli. Resting-state functional connectivity, cerebral metabolism, and perturbational measures provide additional information regarding the integrity and complexity of large-scale brain networks. Although these modalities capture distinct aspects of brain function and are not interchangeable, they offer complementary evidence that contributes to a more comprehensive assessment of consciousness.

Accordingly, the focus of DoC assessment has progressively expanded from determining whether patients can produce observable behavioral responses to evaluating whether their brains retain neural processes associated with consciousness. However, multimodal neurotechnologies do not directly measure consciousness itself. Rather, they provide indirect evidence derived from neural activity, the interpretation of which remains dependent on experimental paradigms, signal quality, acquisition parameters, and analytical methods. Consequently, the observational uncertainty arising from limited behavioral output has not been eliminated but has instead been partially transformed into measurement uncertainty associated with the acquisition and interpretation of neural signals.

### 3.2 Expanding consciousness-related evidence through different neurophysiological paradigms

Active paradigms provide some of the strongest evidence for preserved cognitive function in behaviorally unresponsive patients. Using fMRI, Monti and colleagues demonstrated that a subset of patients who exhibited no observable behavioral responses were nevertheless able to perform mental imagery tasks, such as motor imagery and spatial navigation, in response to verbal commands. Furthermore, distinct patterns of brain activity were successfully used to support simple yes-no communication (17). Similar active paradigms have subsequently been implemented using EEG, yielding evidence of CMD in a subset of patients that is broadly consistent with findings from fMRI studies. Collectively, these studies suggest that some patients who are unable to generate reliable motor output may nevertheless retain command comprehension, sustained attention, and goal-directed information processing, thereby reducing the likelihood of false-negative diagnoses resulting from severe motor impairment.

However, active paradigms still require patients to maintain an adequate level of arousal and to comprehend and follow experimental instructions. Consequently, increasing attention has been directed toward passive paradigms, which elicit neural responses through auditory, emotional, or naturalistic stimuli without requiring active task engagement. Studies have shown that some behaviorally unresponsive patients retain higher-order processing of linguistic and emotionally salient information despite the absence of overt behavioral responses (18). More recently, naturalistic paradigms, such as movie-viewing, have been used to identify neural activity patterns comparable to those observed in healthy individuals, providing complementary evidence of preserved cognitive function that may not be detected using active paradigms alone (19).

Meanwhile, analyses of functional connectivity have demonstrated that the integrity of large-scale brain networks, particularly the default mode network (DMN) and the frontoparietal network, is closely associated with the level of consciousness in patients with DoC (20–22). These findings suggest that DoC is characterized not only by dysfunction within isolated brain regions but also by impaired integration across distributed brain networks. TMS-EEG provides complementary evidence by actively perturbing the cerebral cortex and characterizing the complexity, spatial propagation, and integration of cortical responses. Likewise, FDG-PET studies have shown that a subset of patients diagnosed with UWS based on behavioral assessment exhibit cerebral metabolic patterns more similar to those observed in patients with MCS. These patients have been described as having non-behavioral MCS, reported to occur in approximately 10–15% of the UWS population. However, the definition and diagnostic criteria of this entity remain incompletely established, and it should not be regarded as synonymous with CMD, covert consciousness, or other forms of neurobehavioral dissociation (23, 24). Collectively, these approaches reveal consciousness-related neural evidence that cannot be captured through behavioral assessment alone. Taken together, these findings indicate that the assessment of consciousness has gradually evolved from identifying activation in isolated brain regions to evaluating whether large-scale brain networks retain the capacity to support integrated information processing associated with consciousness.

### 3.3 Multimodal integration and its measurement limitations

The evolution of multimodal assessment represents not only an expansion of available diagnostic techniques but also a fundamental shift in the conceptual approach to consciousness assessment. Rather than relying on multiple technologies in parallel, contemporary multimodal assessment increasingly integrates behavioral assessment with neuroimaging, neurophysiological findings, and clinical information to construct a more comprehensive understanding of the patient’s state. These complementary sources of evidence capture different dimensions of brain function and disease, including behavioral responsiveness, neural activity, large-scale network integrity, and the temporal evolution of neurological injury. By integrating evidence across modalities, clinicians can reduce the risk of misdiagnosis associated with any single assessment method and improve the overall reliability of diagnostic evaluation.

Nevertheless, multimodal assessment also introduces new sources of measurement uncertainty. Variability in imaging equipment, acquisition parameters, experimental paradigms, preprocessing pipelines, and statistical thresholds across centers may compromise the reproducibility and comparability of findings. In addition, patient-related factors-including skull defects, motion artifacts, medication effects, lesion location, and sensory impairments-may degrade signal quality or alter task-related neural responses, thereby affecting the reliability of neurophysiological measurements.

Although multimodal approaches reduce the observational uncertainty associated with relying exclusively on behavioral responsiveness, they do not provide definitive conclusions regarding consciousness. The transition from neural measurement to consciousness inference illustrates the redistribution, transformation, and propagation of uncertainty. By incorporating multiple sources of neural evidence, multimodal assessment reduces dependence on any single behavioral or neurophysiological measure, but redistributes the evidential burden toward establishing measurement reliability and integrating and comparing findings across modalities. As detected neural activity is interpreted as evidence relevant to consciousness, uncertainty is also transformed: the question is no longer only whether a neural response can be detected, but what clinicians may infer about consciousness from that response. At the same time, measurement uncertainty is propagated rather than left behind, because variability in paradigms, acquisition parameters, signal quality, analytical procedures, and patient-related factors continues to constrain the confidence that can be placed in subsequent consciousness attribution. Thus, the transition from neural detection to consciousness inference does not replace measurement uncertainty with an entirely separate form of uncertainty; it reconfigures and carries forward unresolved measurement limitations as neural evidence is translated into claims about consciousness.

## 4 From neural detection to consciousness attribution

### 4.1 Limitations of neural activity as a marker of consciousness

Multimodal neuroimaging and neurophysiological techniques have reduced the reliance of traditional behavioral assessments on motor responsiveness by enabling the direct detection of brain activity, and have increasingly become important tools for identifying residual consciousness in patients with DoC. However, this assessment paradigm is based on an implicit assumption that neural activity can serve as an indirect indicator of conscious awareness (25, 26). Importantly, multimodal techniques detect neural processes associated with consciousness rather than consciousness itself.

A key reason for this limitation is that the interpretation of neural activity requires distinguishing between active command-following responses and passive information processing. The latter is not unique to conscious awareness, whereas reproducible, task-specific responses to active commands provide substantially stronger evidence for inferring consciousness (27, 28). Studies have shown that semantic processing, emotional processing, and stimulus discrimination can persist in the absence of consciousness, generating neural activity detectable by neuroimaging and electrophysiological techniques (29). Evidence from sleep and anesthesia further demonstrates that the brain can retain a certain capacity for processing and integrating external information, producing decodable neural signals even in the absence of explicit subjective awareness (29, 30).

A similar dissociation has been observed in patients with DoC. Behavioral assessments and neural markers do not always converge: some patients who exhibit behavioral evidence of consciousness show only limited neural responses, whereas others who remain behaviorally unresponsive display complex patterns of brain activity (28). These findings indicate that neural activity, behavioral responsiveness, and conscious awareness are not linked by a stable one-to-one correspondence. Consequently, although multimodal techniques increase the likelihood of detecting consciousness-related neural evidence, they cannot establish the presence of consciousness directly. Rather, different types of neural evidence provide different levels of inferential support for consciousness and therefore require interpretation in light of paradigm specificity, reproducibility, and the broader clinical context.

### 4.2 Challenges in interpreting neural evidence

In DoC assessment, uncertainty has shifted from the detection of neural activity to the interpretation of neural evidence. A central challenge lies in the asymmetry between the interpretation of positive and negative findings. Unlike most diagnostic tests in medicine, a positive finding in DoC assessment is generally regarded as important evidence supporting the presence of consciousness, whereas a negative finding cannot be interpreted as evidence of its absence (27). The failure to detect an expected neural response may reflect a variety of factors unrelated to consciousness itself, including impaired attention, deficits in language comprehension, sensory pathway dysfunction, fatigue, sedative medication, or technical limitations and measurement noise (32). Consequently, negative findings cannot be used to exclude the possibility of residual consciousness.

Conversely, even when task-related brain activity is detected, its clinical significance requires careful interpretation. Studies have shown that task-related neural activation may occur even when participants have not fully understood the instructions, indicating that brain activation alone cannot be taken as definitive evidence that a patient comprehended the task or intentionally complied with the command (32). This challenge is particularly evident in studies of CMD. A multicenter study reported that approximately 25% of behaviorally unresponsive patients demonstrated command-following on fMRI or EEG. Conversely, among patients who exhibited behavioral command-following at the bedside, the majority failed to show positive findings on at least one active neuroimaging paradigm (31). These observations highlight that multimodal techniques reduce the uncertainty arising from the inability to detect neural activity, but simultaneously introduce a new source of uncertainty concerning what detected neural activity actually signifies. Accordingly, positive findings from active paradigms substantially increase the likelihood of consciousness, whereas negative findings generally provide only limited evidence against its presence. As a result, the central challenge in DoC assessment has shifted from determining whether neural activity is present to interpreting what that neural evidence implies for consciousness.

### 4.3 Epistemological limits of measuring consciousness

Although multimodal neuroimaging and neurophysiological techniques provide increasingly rich neural evidence, they cannot, by themselves, establish whether a patient with DoC is consciously aware. Unlike physiological variables such as blood pressure or blood glucose, which can be directly measured by external observers, consciousness is fundamentally a first-person subjective experience that is directly accessible only to the experiencing individual. As Kirkeby-Hinrup et al. (33) argue, the study of consciousness ultimately depends on first-person reports as the reference standard for validating conscious experience. In patients with DoC, however, this crucial source of validation is unavailable because of impaired behavioral expression. Consequently, clinicians and researchers must infer the presence of consciousness indirectly from external indicators-including behavioral performance, neural activity, and brain network characteristics-rather than observe consciousness itself.

Even when robust neural evidence is available, its significance remains dependent on the theoretical framework through which it is interpreted. Different theories of consciousness offer distinct accounts of which neural processes constitute reliable indicators of conscious awareness. For example, the Global Neuronal Workspace Theory emphasizes widespread information broadcasting, whereas the Integrated Information Theory focuses on the degree of information integration within the system (34, 35). Consequently, neural evidence is not self-interpreting; rather, its clinical significance is theory-dependent and can only be understood within a specific conceptual framework of consciousness.

Taken together, multimodal techniques have transformed the way consciousness-related evidence is acquired, but they have not eliminated the inherently inferential nature of consciousness assessment. Accordingly, uncertainty extends beyond the detection of neural activity to the interpretation of neural evidence and, ultimately, to the attribution of consciousness itself (36). This transition illustrates the transformation and propagation of uncertainty. Once detected neural activity is used to support consciousness attribution, the question changes from whether a neural response can be reliably identified to whether that response provides sufficient grounds for inferring conscious awareness. Measurement limitations are carried forward into this process because paradigm specificity and signal reliability continue to constrain the strength of the resulting inference. Moreover, the asymmetrical interpretation of positive and negative findings, discordance between behavioral and neural evidence, and theoretical disagreement about neural indicators of consciousness may coexist, allowing inferential uncertainty to accumulate rather than being resolved by any single finding. In the absence of direct first-person validation, this unresolved uncertainty is subsequently carried into prognostic evaluation and clinical decision-making.

## 5 From consciousness attribution to clinical decision-making

### 5.1 Challenges in prognostic decision-making

Although multimodal techniques have improved the detection of consciousness, they have not eliminated the uncertainty surrounding the interpretation of neural evidence. Clinical practice, however, cannot be suspended because evidence remains inconclusive. Clinicians must still decide whether to continue life-sustaining treatment, when to initiate rehabilitation, or whether to shift the goals of care. Consequently, the central challenge of prognostic assessment is not to achieve absolute certainty, but to make consequential clinical decisions on the basis of inherently probabilistic evidence. This structural tension between uncertain evidence and unavoidable decisions constitutes a fundamental challenge in prognostic assessment for patients with DoC.

Against this background, the phenomenon of the self-fulfilling prophecy has emerged as one of the most important concerns in neurocritical care. When clinicians limit treatment or withdraw life-sustaining treatment on the basis of an early pessimistic prognosis, patients who might otherwise have recovered may lose the opportunity for meaningful neurological improvement, thereby allowing the initial prognostic judgment to become self-fulfilling (37). To reduce this risk, an advisory statement for comatose survivors of cardiac arrest recommends a multimodal prognostication approach and prolonged observation with repeated assessments when initial findings are inconclusive (38). In addition, multidisciplinary discussions, the involvement of surrogate decision-makers, and time-limited trials of treatment may help preserve opportunities for further observation and neurological recovery when prognostic uncertainty remains substantial (39, 40).

Importantly, the presence of consciousness and functional prognosis represent distinct clinical questions (41). Although multimodal techniques can identify residual consciousness in some patients who would otherwise be missed by behavioral assessment, evidence of consciousness does not necessarily imply the potential for meaningful functional recovery. Current evidence suggests that functional neuroimaging and other neural biomarkers remain limited in their ability to predict long-term functional outcomes (42). In essence, consciousness assessment addresses whether a patient is likely to retain conscious awareness at the present time, whereas prognostic assessment seeks to estimate the degree of future functional recovery. Although these two domains are closely related, they should not be regarded as interchangeable.

The transition from consciousness attribution to functional prognostication illustrates the propagation and transformation of uncertainty. Inferential uncertainty concerning the patient’s current state is carried forward because the reliability of consciousness attribution constrains the evidential basis of subsequent prognosis. At the same time, uncertainty changes in form as the clinical question moves from whether conscious awareness is presently retained to what degree of functional recovery may occur in the future. Evidence that reduces uncertainty regarding current consciousness may therefore leave prognostic uncertainty substantially unresolved.

Therefore, the goal of prognostic assessment in DoC is not to achieve absolute predictive certainty, but to support responsible clinical decision-making while explicitly acknowledging persistent uncertainty. This requires delaying prognostication when appropriate, integrating multimodal evidence, conducting longitudinal reassessments, and adopting a cautious approach to irreversible treatment decisions. In doing so, clinicians can reduce the risk of prematurely translating probabilistic evidence into irreversible clinical outcomes. More broadly, this shift-from pursuing certainty to managing uncertainty-represents a fundamental change in the philosophy of clinical decision-making for patients with DoC.

### 5.2 Value deliberation in surrogate decision-making

For most patients with DoC, the inability to communicate their own preferences means that the diagnostic and prognostic uncertainties inherent in clinical assessment must ultimately be translated into treatment decisions through surrogate decision-making. When medical evidence cannot provide a single definitive answer, surrogate decision-makers must not only interpret probabilistic clinical information but also weigh the patient’s previously expressed values, family expectations, and the potential benefits and burdens of treatment (43). At this stage, uncertainty is no longer solely a medical issue; rather, it evolves into a multidimensional challenge in which clinical evidence, personal values, and ethical considerations become inseparably intertwined.

Disagreements in surrogate decision-making do not arise solely from insufficient information. Studies have shown that family members and healthcare professionals may reach substantially different conclusions about the likelihood of recovery even when they are presented with the same clinical evidence (44). Moreover, differences in values further amplify the consequences of these divergent interpretations. Family members often regard access to advanced neurotechnologies as an ethical obligation to maximize every possible opportunity for recovery, whereas clinicians are more likely to balance the potential benefits of these technologies against their clinical limitations and burdens (45). Consequently, disagreements in surrogate decision-making reflect not only different interpretations of the facts but also different value frameworks through which the same evidence is understood. At this stage, the central question shifts from whether the patient is conscious to how clinicians and families ought to act in the face of that possibility.

Furthermore, professional recommendations themselves cannot be entirely value-neutral. As Graham (42) has argued, clinical recommendations regarding life-sustaining treatment, rehabilitation goals, and quality of life often implicitly incorporate value judgments about acceptable outcomes, functional recovery, and the meaning of treatment. Accordingly, when uncertainty persists, clinicians should not only explain the available medical evidence but also make explicit the normative assumptions underlying their recommendations. Doing so enables families to better understand the ethical implications of different options and to participate more meaningfully in shared decision-making.

Taken together, the transition from consciousness attribution and prognostic evaluation to surrogate decision-making illustrates the propagation, transformation, and redistribution of uncertainty. Unresolved diagnostic and prognostic uncertainty is propagated into treatment decisions because the need to act remains even when the available evidence is inconclusive. As probabilistic clinical evidence is considered alongside the patient’s values, family expectations, and the anticipated benefits and burdens of treatment, uncertainty is transformed from an epistemic question about what is known into a normative question about what ought to be done. Responsibility for interpreting and managing this uncertainty is also redistributed across clinicians and surrogate decision-makers through communication and shared deliberation. When uncertainty in clinical evidence interacts with divergent interpretations and value frameworks, epistemic and normative uncertainties may accumulate and contribute to persistent disagreement rather than yielding a single decision determined by medical evidence alone.

### 5.3 Algorithmic uncertainty and AI governance

AI should not be understood solely as a downstream layer between the interpretation of neural evidence and clinical decision-making. Across the DoC assessment pathway, computational approaches are increasingly being explored for neural signal processing and classification, multimodal data integration, consciousness-state inference, prognostic prediction, and clinical decision support (46, 47). AI-related uncertainty should therefore be conceptualized as a cross-cutting layer that interacts with measurement, inference, prognosis, and decision-making rather than as a separate form of uncertainty confined to the final stage. Although AI has considerable potential to integrate heterogeneous data and improve predictive performance, it does not eliminate the uncertainties already embedded in DoC assessment. Instead, AI models may inherit uncertainties from their input data, proxy measures, and clinical labels while introducing additional uncertainties related to model development, validation, generalizability, and output interpretation.

AI models proposed in DoC-related research generally do not identify consciousness directly. Instead, they generate classifications or predictions from proxy measures, which may include behavioral assessment scores, electroencephalographic features, functional neuroimaging findings, or clinically defined outcome labels (48, 49). Because these inputs and targets are themselves indirect and potentially imperfect representations of consciousness or recovery, algorithmic outputs may inherit uncertainties already present in behavioral assessment, neural measurement, and clinical classification. More generally, Obermeyer et al. (50) demonstrated that bias in healthcare algorithms may arise from the selection of an inadequate proxy target rather than from model architecture alone. Although their analysis was not specific to DoC, the underlying concern is relevant here: if the variables used to train or evaluate an AI model do not adequately represent the patient state or outcome of interest, high statistical performance does not necessarily establish clinical or conceptual validity. AI may therefore propagate systematic errors embedded in existing assessment frameworks and, under some conditions, amplify them.

Explainable artificial intelligence (XAI) does not by itself resolve these concerns. Ghassemi et al. (51) cautioned that current XAI approaches may describe how particular features contribute to a model’s output without establishing whether those features are clinically meaningful or valid representations of the target phenomenon. Although their argument was not specific to DoC, it highlights an important limitation in this context. A transparent account of how an AI system produces a classification or prediction cannot determine whether the behavioral, electrophysiological, or neuroimaging features on which it relies provide valid evidence of consciousness. Explainability may therefore clarify model operation, but it does not eliminate the measurement and inferential uncertainties embedded in the model. If an explanation is treated as evidence that the prediction itself is valid, it may instead create unwarranted confidence in an unreliable output.

Taken together, AI does not represent a final stage to which uncertainty is transferred after clinical evidence has been interpreted. Rather, its interaction with multiple stages of the assessment pathway allows existing uncertainty to be propagated while additional algorithmic uncertainty is introduced (46, 47). Progress in this field should therefore be evaluated in relation to both predictive performance and governance. Model inputs, training targets, validation procedures, intended applications, and output limitations should be transparent and auditable. Governance arrangements should also clarify professional accountability and protect patients’ values and interests (52). Ultimately, AI should serve as a tool to support clinical judgment rather than become an independent source of clinical authority or create an illusion of certainty.

## 6 Conclusion

The evolution of DoC assessment reflects an expansion from behavioral observation to the incorporation of multimodal neural evidence. Although multimodal techniques have reduced some of the observational uncertainty arising from impaired motor output and fluctuations in behavioral responsiveness, they may also introduce sources of measurement uncertainty related to paradigm dependence, signal quality, and methodological variability. When neural findings are used to infer the presence of consciousness, measurement uncertainty may persist and be transformed into uncertainty concerning evidence interpretation and consciousness attribution. As consciousness attribution informs prognostic evaluation and surrogate decision-making, unresolved uncertainty may propagate into prognostic and normative domains. Technological advances may also redistribute the remaining uncertainty toward evidence interpretation and clinical judgment, while residual and newly introduced uncertainties may accumulate across successive stages. Meanwhile,algorithmic uncertainty, meanwhile, may arise across the assessment pathway wherever AI methods are used to process evidence, generate predictions, or support clinical decisions. Consequently, technological advances have fundamentally transformed the acquisition of consciousness-related evidence, but they have neither eliminated the inherently inferential nature of consciousness assessment nor necessarily produced more definitive clinical conclusions (4, 5).

Against this background, the goal of DoC assessment should extend beyond improving diagnostic sensitivity to recognizing, stratifying, and managing uncertainty throughout the assessment process. In clinical practice, repeated, longitudinal, and multimodal assessments should be prioritized, and different types of neural evidence should be interpreted according to their task specificity, reproducibility, and clinical context to reflect their differing inferential strength (53). When discrepancies arise between behavioral and neurophysiological findings, or across different neuroimaging modalities, they should not be forced into a single definitive conclusion. Instead, such inconsistencies should be explicitly documented together with their potential sources. At the same time, consciousness assessment, functional prognostication, and treatment-related value judgments should remain conceptually distinct. Evidence supporting the presence of consciousness should not be equated with evidence of favorable long-term functional recovery, nor should an unfavorable prognosis be prematurely translated into irreversible treatment decisions (41, 42).

Shared decision-making and transparent governance constitute essential strategies for managing persistent uncertainty in DoC care. Clinicians should communicate findings to families in probabilistic terms, explicitly acknowledging the limits of available evidence while clearly distinguishing empirical medical judgments from value-based considerations. Likewise, AI models should be regarded as decision-support tools rather than independent decision-makers. Their training labels, external validation, intended scope of application, and the uncertainty associated with their outputs should all be subject to transparent evaluation and ongoing oversight (50–52). Ultimately, the goal of DoC assessment should not be to pursue absolute certainty, but to support more cautious, transparent, and patient-centered clinical decision-making while explicitly recognizing the limits of current evidence.
